# The value of transpulmonary thermodilution parameters in predicting hemodynamic instability in intensive care patients undergoing continuous renal replacement therapy

**DOI:** 10.1186/s12882-025-04712-0

**Published:** 2025-12-20

**Authors:** Cagla Sena Keser, Mete Erdemir, Mahmut Yilmaz, Gurhan Taskin, Levent Yamanel

**Affiliations:** 1 Department of Internal Medicine, Gulhane Training and Research Hospital, Ankara, Türkiye; 2 Department of Intensive Care, Gulhane Training and Research Hospital, Gen. Dr. Tevfik Sağlam Street No:1, Keçiören, Ankara, 06010 Türkiye

**Keywords:** Acute kidney injury, Continuous renal replacement therapy, Hemodynamics, Intensive care units, Cardiac output, Vascular resistance

## Abstract

**Background:**

Hemodynamic instability related to renal replacement therapy (HIRRT) is a serious complication of continuous renal replacement therapy (CRRT) in critically ill patients with acute kidney injury (AKI), significantly increasing mortality risk. The pathophysiology involves complex interactions between cardiac output and systemic vascular resistance. Therefore, identifying early and reliable predictive parameters for HIRRT is clinically crucial.

**Methods:**

This prospective, observational cohort study was conducted in an Internal Medicine Intensive Care Unit between July 2023 and October 2024. Thirty-six patients undergoing CRRT with invasive monitoring via the PiCCO^®^ device were enrolled. Hemodynamic parameters were recorded. HIRRT was defined exclusively as a decrease in systolic blood pressure ≥ 20 mmHg or a decrease in mean arterial pressure ≥ 10 mmHg. Changes in vasopressor or inotropic therapy were not included in the diagnostic criteria for HIRRT. To avoid classifying transient or clinically insignificant fluctuations as HIRRT, the decrease in blood pressure had to be present in at least two consecutive measurements (approximately 10–15 min). This definition is consistent with prior CRRT studies that use objective blood pressure thresholds. Statistical analyses included the Mann-Whitney U test, chi-square test, logistic regression, and receiver operating characteristic (ROC) curve analysis.

**Results:**

The mean age of the patients was 68.1 ± 18.3 years, and 69.4% were male. The most common indication for CRRT was uremic complications (52.8%), and the most frequent comorbidity was hypertension (55.6%). A total of 83.3% of patients were on vasopressor support, and 38.9% were on mechanical ventilation. HIRRT occurred in 55.5% of patients during CRRT, and in 60% of these patients, it developed within the first hour. The HIRRT group had a significantly lower cardiac index (CI) (*p* = 0.002) and a higher systemic vascular resistance index (SVRI) (*p* = 0.003). Additionally, the HIRRT group had higher baseline mean arterial pressure (*p* = 0.015) and baseline diastolic blood pressure (*p* = 0.003), and a significantly greater total ultrafiltration volume (*p* = 0.018). Multivariate analysis identified a low CI (*p* = 0.018) and a high mean arterial pressure (*p* = 0.031) as independent predictors. ROC analysis revealed that the optimal cut-off value for the mean arterial pressure was 78 mmHg (AUC: 0.759, 95% CI: 0.597–0.922) and that for the CI was 2.61 L/min/m² (AUC: 0.794, 95% CI: 0.642–0.946).

**Conclusions:**

Low baseline cardiac index and elevated mean arterial pressure emerged as independent predictors of HIRRT, whereas higher SVRI was significant only in univariate analysis, indicating an association but not independent predictive value. Collectively, these findings suggest that a reduced capacity to increase cardiac output in response to hemodynamic stress, together with alterations in vascular tone regulation, may play a central role in the development of HIRRT. While these parameters may facilitate early identification of high-risk patients and support individualized management strategies, further validation in larger, multicenter cohorts is required.

**Clinical trial number:**

Not applicable.

## Introduction

Acute kidney injury (AKI) is a serious clinical condition that significantly increases morbidity and mortality in intensive care units (ICUs) and often requires continuous renal replacement therapy (CRRT) in its advanced stages [1]. CRRT is a preferred treatment method, particularly for patients with hemodynamic instability [1]. However, hemodynamic instability related to renal replacement therapy (HIRRT) is considered one of the most serious complications of CRRT, occurring in 20–50% of patients and increasing mortality risk [2]. The pathophysiology of HIRRT involves hypovolemia, decreased cardiac output, and changes in systemic vascular resistance as key mechanisms [3].

Although there is no universally accepted definition for HIRRT, the literature commonly uses diagnostic criteria such as a decrease in systolic blood pressure of ≥ 20 mmHg or a decrease in mean arterial pressure of ≥ 10 mmHg, the presence of symptoms, or situations requiring medical intervention [4, 5]. In line with the literature, this study adopted the criteria of a ≥ 20 mmHg drop in systolic blood pressure or a ≥ 10 mmHg decrease in mean arterial pressure [6, 7].

Transpulmonary thermodilution (TPTD) is an invasive hemodynamic monitoring method used to assess cardiac output and volume status [8]. This technique provides parameters such as the global end-diastolic volume index (GEDI) and the extravascular lung water index (ELWI) [9]. However, the role of these parameters in predicting HIRRT has not been sufficiently investigated.

This study examined the relationship between TPTD parameters and the development of HIRRT in patients receiving CRRT. These findings may contribute to the early identification of patients at high risk for hemodynamic instability and the individualization of treatment strategies.

## Materials and methods

### Study design and ethical approval

This prospective, observational cohort study was conducted in the Intensive Care Unit of Gülhane Training and Research Hospital between July 2023 and October 2024. The study was approved by the Gülhane Training and Research Hospital Clinical Research Ethics Committee (Protocol No: 2023/203, September 27, 2023) and was carried out in accordance with the Helsinki Declaration. Following ethics approval, all consecutive eligible patients undergoing CRRT with PiCCO monitoring were prospectively enrolled throughout the entire study period (July 2023–October 2024). Written informed consent was obtained from the first-degree relatives of all participants. No formal sample size calculation was performed, as this was an exploratory, observational cohort study designed to include all consecutive eligible patients within the defined study period. The study therefore reflects the full available population rather than a predetermined sample size based on statistical hypotheses.

### Study population

#### Inclusion criteria

1. Patients aged 18 years or older who provided informed consent, either personally or through a legally authorized representative.
2. Patients undergoing invasive hemodynamic monitoring with the PiCCO^®^ device (Pulsion Medical Systems, Germany).
3. Patients with an indication for CRRT due to acute or chronic renal failure.

#### Exclusion criteria

1. Patients who did not provide consent.
2. Patients under 18 years of age.
3. Pregnant patients.
4. Patients with terminal-stage malignancy.
5. Patients who had clinical conditions known to compromise the reliability of transpulmonary thermodilution measurements (e.g., intracardiac shunts, severe tricuspid regurgitation, severe aortic valve insufficiency, or massive pulmonary embolism).

### Data collection and monitoring protocol

The demographic characteristics, comorbidities, laboratory findings, and treatment parameters of all enrolled patients were prospectively recorded. Hemodynamic parameters (cardiac index [CI], systemic vascular resistance index [SVRI], stroke volume variation [SVV], pulse pressure variation [PPV], GEDI, and ELWI) were measured using the PiCCO^®^ device at hourly intervals before and during CRRT. SVRI was calculated by the PiCCO^®^ system using the standard formula incorporating the MAP–CVP gradient, and CVP values obtained from the central venous catheter were entered at the time of transpulmonary thermodilution measurements. Baseline hemodynamic parameters were defined as the measurements obtained immediately before CRRT initiation. In patients with spontaneous breathing activity, PPV and SVV were automatically recorded by the PiCCO^®^ system but were not considered physiologically interpretable.

### Continuous renal replacement therapy protocol

CRRT was administered to all patients using Multifiltrate/MultifiltratePro machines (Fresenius Medical Care, Bad Homburg, Germany) and Multibic^®^ potassium-free dialysis solutions, in accordance with the standard hospital protocol. The anticoagulation rate, ultrafiltration rate, and dialysate flow rate were determined individually for each patient via titration.

### Study endpoints

#### Primary endpoint

The primary endpoint of the study was the development of HIRRT, defined according to the criteria described above.

#### Secondary endpoints

Secondary endpoints included the identification of baseline hemodynamic parameters (CI, SVRI, MAP, GEDI, ELWI, SVV, and PPV) associated with the development of HIRRT, as well as the comparison of baseline hemodynamic characteristics between patients who developed early HIRRT (within the first hour of CRRT) and those who did not.

### Statistical analysis

Data analysis was performed using IBM SPSS Statistics version 27.0 (IBM Corp., Armonk, NY, USA). In descriptive statistics, continuous variables are presented as mean ± standard deviation or median (interquartile range), based on their conformity to a normal distribution. Categorical variables are expressed as numbers and percentages. For intergroup comparisons, the Chi-square or Fisher’s Exact test was used for categorical data, and non-parametric tests (Mann-Whitney U, Kruskal-Wallis) were applied for continuous data. The Spearman correlation coefficient was calculated for correlation analysis. The diagnostic performance of hemodynamic parameters in predicting HIRRT was evaluated using Receiver Operating Characteristic (ROC) curve analysis, with optimal cut-off points determined by the Youden index. Multivariate logistic regression analysis was performed to identify risk factors. The relationship between SVRI values and HIRRT was examined by dividing SVRI into three groups based on the interquartile range (IQR). A p-value of < 0.05 was considered statistically significant. Given the limited number of HIRRT events (*n* = 20), the multivariable logistic regression model was restricted to a maximum of two predictors in accordance with the commonly applied rule of approximately 10 events per variable. CI and baseline mean arterial pressure (MAP) were selected a priori based on strong physiological plausibility and their significance in univariable analyses. Logistic regression results are presented as odds ratios (ORs) with 95% confidence intervals (CIs).

## Results

**Fig. 1:**
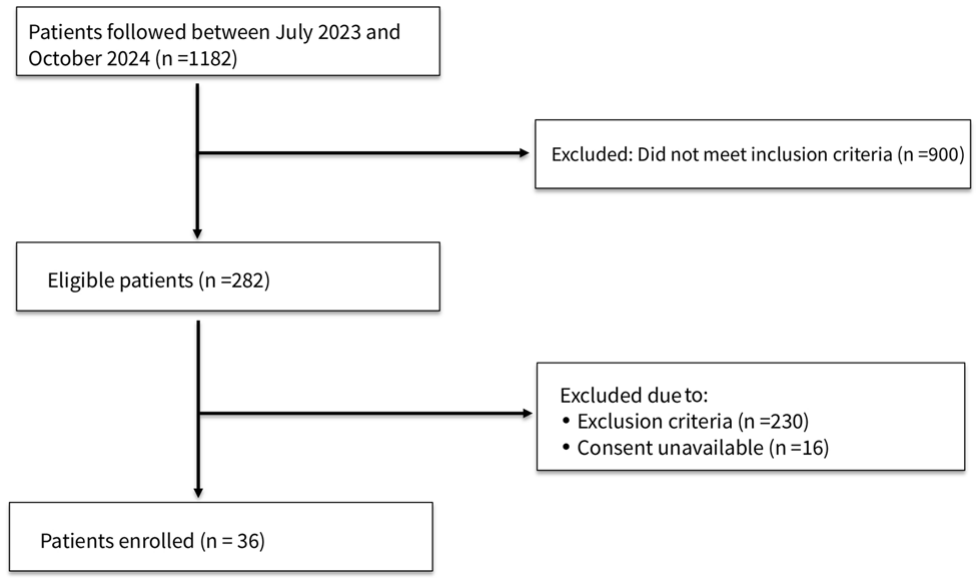
Study flowchart

A total of 36 patients were included in the study (Fig. 1). The mean age was 68.1 ± 18.28 years (range: 23–91), with 69.4% (*n* = 25) being male. The most prevalent comorbidities were hypertension (55.6%), diabetes mellitus (33.3%), and coronary artery disease (25%). Regarding supportive care, 83.3% (*n* = 30) of the patients required vasopressor support and 38.9% (*n* = 14) were on mechanical ventilation.

Regarding CRRT indications, the most common indication was uremic complications (52.8%, *n* = 19), followed by hypervolemia (22.2%, *n* = 8), severe metabolic acidosis (11.1%, *n* = 4), hyperkalemia (8.3%, *n* = 3), and chronic kidney disease (5.6%, *n* = 2). Evaluation of the reasons for CRRT discontinuation revealed that treatment was stopped because the therapeutic goal was achieved in 50% (*n* = 18) of the patients. Other primary reasons for discontinuation were hyperlactatemia (22.2%, *n* = 8), hemodynamic instability (13.9%, *n* = 5), and technical issues (13.9%, *n* = 5). The duration of CRRT ranged from 2 to 36 h, with a mean treatment duration of 18.02 ± 9.92 h. The in-hospital mortality rate in the study cohort was 75%, indicating the high-risk profile of this ICU patient group.

HIRRT developed in 55.5% (*n* = 20) of the patients during CRRT, and in 60% (*n* = 12) of these patients, the instability began within the first hour of treatment (Table 1). HIRRT was not significantly associated with demographic factors, comorbidities, ventilator status, vasopressor use, or CRRT indications.

**Table 1:** Comparison of groups regarding the development of hemodynamic instability during

Variables	HIRRT (+) (*n* = 20)	HIRRT (-) (*n* = 16)	*P*-value
**Demographics**			
Age (Years)	69.9 ± 15.9	65.8 ± 21.2	0.519^a^
Gender			0.067^C^
Male	11 (55)	14 (87.5)	
Female	9 (45)	2 (12.5)	
**Mechanical Ventilation Support**			0.400^C^
Yes	11 (55)	11 (68.8)	
No	9 (45)	5 (31.2)	
**Vasopressor Support**			0.672^c^
Yes	16 (80)	14 (87.5)	
No	4 (20)	2 (12.5)	
**CRRT Duration (h)**	17.3 ± 10.13	18.93 ± 9.9	0.630^a^
**Total Ultrafiltration(mL)**	650 (0-7750)	0 (0-3400)	**0.018** ^b*^
**CRRT Termination Indication**			0.270^d^
Achievement of Target	10 (50)	8 (50)	
Hemodynamic Instability	4 (20)	1 (6.2)	
Technical Reasons	1 (5)	4 (25)	
Hyperlactatemia	5 (25)	3 (18.8)	
**Comorbidites**			
Diabetes Mellitus	8 (40)	4 (25)	0.481^c^
Hypertension	13 (65)	7 (43.8)	0.202^d^
Coronary Artery Disease	4 (20)	5 (31.3)	0.470^c^
Heart Failure	5 (25)	2 (12.5)	0.426^c^
Malignancy	2 (10)	5 (31.3)	0.204^c^
Cerebrovascular Event	4 (20)	1 (6.3)	0.355^c^
Chronic Obstructive Disease	3 (15)	4 (25)	0.675^c^
Chronic Kidney Disease	6 (30)	1 (6.3)	0.104^c^
**Hemodynamic Parameters (PiCCO)**			
Heart Rate (beats/min)	96.15 ± 21.47	90.56 ± 11.36	0.354^a^
SBP (mmHg)	116.35 ± 22.07	109.75 ± 17.39	0.336^a^
DBP (mmHg)	69.40 ± 9.57	57.56 ± 2.81	**0.003** ^**a**^
MAP (mmHg)	85.06 ± 12.72	74.91 ± 2.64	**0.015** ^**a**^
CI (L/dk/m^2)^	2.24 ± 0.69	3.23 ± 1.06	**0.002** ^**a**^
CPI (watt/m^2^)	195.11 ± 84.94	239.96 ± 79.64	0.115^a^
GEDI (mL/m^2)^	675(363–1019)	744.5 (334–1424)	0.140^b^
ELWI (ml/kg)	8 (4–18)	8.59 (4–40)	0.962^b^
SVRI (dyn, s·cm⁻⁵·m²)	2572 (1976–5773)	1922 (856–3569)	**0.003** ^**b**^
SVRI Groups n(%)			**0.002** ^**d**^
Normal	6 (30)	2 (12.5)	
Low	0 (0)	8 (50)	
High	14 (70)	6 (37.5)	
SVV(*n* = 25)	13 (6–29)	15.5 (3–29)	0.313^b^
PPV (*n* = 32)	13.5 (4–26)	17 (3–27)	0.473^b^
**Baseline Laboratory Values**			
pH	7.35 (7.03–7.46)	7.41 (6.84–7.49)	0.276^b^
PaCO_2_ (mmHg)	38.47 ± 8.89	36.76 ± 11.75	0.623^a^
HCO_3_^−^ (mEq/L)	20.47 ± 5.63	21.43 ± 8.49	0.685^a^
Lactate (mmol/L)	2.45 (0.8–11.7)	2.5 (1-14.6)	0.962^b^
Creatinine (mg/dL)	3.59 (1.63–8.47)	2.98 (1.44–10.02)	0.320^b^
Urea (mg/dL)	192.6 ± 85.87	189.95 ± 61.58	0.918^a^
Na^+^(mEq/L)	142.65 ± 10.39	141.06 ± 13.97	0.698^a^
K^+^(mEq/L)	4.1 (3.2–5.6)	3.95 (3-7.2)	0.987^b^
**In-Hospital Mortality**	16 (80)	11 (68.8)	0.470^c^

CRRT, Continuous Renal Replacement Therapy; UF, Ultrafiltration; SBP, Systolic Blood Pressure; DBP, Diastolic Blood Pressure; MAP, Mean Arterial Pressure; CI, Cardiac Index; CPI, Cardiac Pressure Index; GEDI, Global End-Diastolic Volume Index; ELWI, Extravascular Lung Water Index; SVRI, Systemic Vascular Resistance Index; PaCO₂, Partial Pressure of Arterial Carbon Dioxide; HCO₃⁻. Bicarbonate

Data are presented as n (%), mean ± standard deviation, or median (min-max). a, Student’s t-test; b, Mann-Whitney U test; c, Fisher’s Exact test; d, Pearson Chi-square test

Analysis of hemodynamic parameters revealed a significantly lower CI (2.24 ± 0.69 vs. 3.23 ± 1.06; *p* = 0.002) and a higher Systemic Vascular Resistance Index (SVRI) (2572 [1976–5773] vs. 1922 [856–3569]; *p* = 0.003) in the group that developed HIRRT. In contrast, there were no significant differences between the groups in terms of ELWI, GEDI, SVV, and PPV values (*p* > 0.05). Furthermore, in the HIRRT group, the baseline mean arterial pressure (85.06 ± 12.72 vs. 74.91 ± 2.64 mmHg; *p* = 0.015) and baseline diastolic blood pressure (69.4 ± 9.57 vs. 57.56 ± 2.81 mmHg; *p* = 0.003) were higher, and the total ultrafiltration volume was also significantly greater (650 [0-7750] vs. 0 [0-3400] mL; *p* = 0.018). All baseline values reported in this analysis correspond to measurements obtained immediately prior to CRRT initiation.

**Fig. 2:**
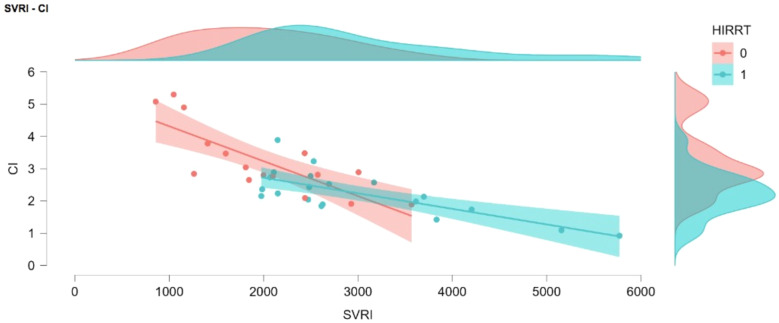
Comparative schematic of cardiac ındex (CI) and systemic vascular resistance ındex (SVRI) values stratified by HIRRT development

**Fig. 3:**
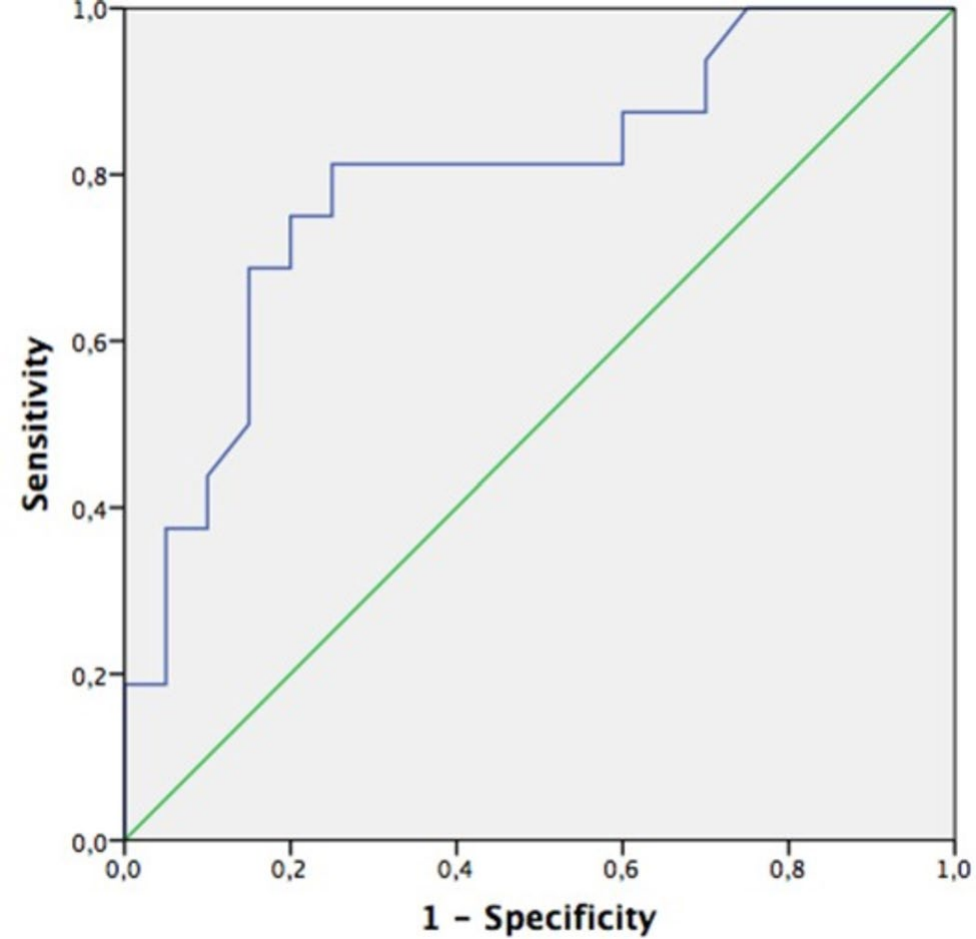
Receiver operating characteristic (ROC) curve analysis of the cardiac ındex for predicting hemodynamic instability related to renal replacement therapy (HIRRT)

In the multivariate logistic regression analysis, lower CI and higher baseline mean arterial pressure were identified as independent predictive factors for the development of HIRRT. CI was independently associated with HIRRT (OR: 0.107, 95% CI: 0.017–0.687; *p* = 0.018), and baseline MAP was also a significant predictor (OR: 1.115, 95% CI: 1.010–1.231; *p* = 0.031). The model explained 61.2% of the variance (R²=0.612). ROC analyses determined the optimal cut-off value for MAP to be 78 mmHg (AUC: 0.759, 95% CI: 0.597–0.922) and for CI to be 2.61 L/min/m² (AUC: 0.794, 95% CI: 0.642–0.946) (Figs. 2 and 3). As an exploratory analysis, we also evaluated a two-variable model including CI and SVRI only; in this model, CI remained independently associated with HIRRT, whereas SVRI did not retain statistical significance after adjustment for CI.

In the group that developed HIRRT within the first hour of CRRT, the baseline CI value was significantly lower (2.06 ± 0.79 vs. 2.99 ± 0.95; *p* = 0.007), and the baseline SVRI value was significantly higher (2619 [2067–5773] vs. 2104 [856–3699]; *p* = 0.014) (Table 2). These findings suggest that early changes in cardiac output and vascular resistance may be determinants in the pathogenesis of HIRRT. These differences were found to be unrelated to vasopressor dose (*p* = 0.135). There were no significant differences between the groups regarding other static hemodynamic parameters (such as systolic and diastolic blood pressure, and heart rate) (Table 2).

**Table 2:** Comparison of patient characteristics and hemodynamic parameters between groups with and without hemodynamic instability related to renal replacement therapy (HIRRT) during the first hour of continuous renal replacement therapy (CRRT)

Variables	HIRRT in first hour (+) (*n* = 12)	HIRRT in first hour (–) (*n* = 24)	*P* value
**Demographics**			
Age (years)	68.41 ± 17.25	67.95 ± 19.13	0.945^a^
Gender			0.073^d^
Male	6 (50)	19 (79.2)	
Female	6 (50)	5 (20.8)	
**Comorbidities**			
Diabetes Mellitus	3 (25)	9 (37.5)	0.709^c^
Hypertension	6 (50)	14 (58.3)	0.729^c^
Coronary Artery Disease	2 (16.7)	7 (29.2)	0.685^c^
Heart Failure	2 (16.7)	5 (20.8)	1.000^c^
Malignancy	2 (16.7)	5 (20.8)	1.000^c^
Cerebrovascular Event	2 (16.7)	3 (12.5)	1.000^c^
Chronic Obstructive Pulmonary Disease	1 (8.3)	6 (25)	0.384^c^
Chronic Kidney Disease	2 (16.7)	5 (20.8)	1.000^c^
**Mechanical Ventilation Support**			0.091^d^
Yes	5 (41.7)	17 (70.8)	
No	7 (58.3)	7 (29.2)	
**Vasopressor Support**			1.000^c^
No	2 (16.7)	4 (16.7)	
Yes	10 (83.3)	20 (83.3)	
**Vasopressor dose at CRRT initiation**	0.25 (0.06–1.25)	0.10 (0.04–1.40)	0.135^b^
**CRRT duration (h)**	12.58 ± 7.26	20.75 ± 10.06	**0.018** ^**a**^
**CRRT Indication**			0.419^d^
Routine CKD dialysis	0	2 (8.3)	
Hyperkalemia	0	3 (12.5)	
Metabolic acidosis	2 (16.7)	2 (8.3)	
Uremic complications	8 (66.7)	11 (45.8)	
Hypervolemia	2 (16.7)	6 (25)	
**Hemodynamic Parameters**			
HR (beats/min)	99 ± 19	91 ± 16.5	0.206 ^a^
SBP (mmHg)	119 ± 27	110 ± 15	0.284 ^a^
DBP (mmHg)	68 ± 11	61 ± 12	0.099 ^a^
MAP (mmHg)	85 ± 15	77 ± 10	0.138 ^a^
CI (L/dk/m^2)^	2.06 ± 0.79	2.99 ± 0.95	**0.007** ^**a**^
CPI (watt/m^2^)	184.32 ± 104.78	230.4 ± 69.89	0.125^a^
GEDI (mL/m^2)^	675 (363–855)	716 (334–1424)	0.115^b^
ELWI (ml/kg)	8 (6–18)	8.5 (4–40)	0.787^b^
SVRI (dyn.s·cm⁻⁵·m²)	2619 (2067–5773)	2104 (856–3699)	**0.014** ^**b**^
SVV	13.5 (6–21)	14 (3–29)	0.661^b^
PPV	13.5 (4–25)	17 (3–27)	0.207^b^
pH	7.35 (7.03–7.46)	7.38 (6.84–7.49)	0.214^b^
**Baseline Laboratory Findings**			
pH	7.35 (7.03–7.46)	7.38 (6.84–7.49)	0.214^b^
PaCO_2_ (mmHg)	40.3 ± 10.1	36.3 ± 10.0	0.269^a^
HCO_3_^−^ (mEq/L)	23 (6.7–29.7)	20.3 (2.2–34.3)	0.933^b^
Lactate (mmol/L)	3.0 (0.8–11.7)	2.3 (0.9–14.9)	0.450^b^
Creatinine (mg/dL)	3.69 (1.65–6.28)	3.04 (1.44–10.02)	0.675^b^
Urea (mg/dL)	204.4 ± 98.9	184.9 ± 61.2	0.541^a^
Na^+^(mEq/L)	146.1 ± 11.2	139.8 ± 11.9	0.136^a^
Potasyum (mEq/L)	4.1 (3.2–5.6)	3.95 (3-7.2)	0.987^b^
**In-hospital Mortality**	10 (%83.3)	17 (%70.8)	0.685^c^

CRRT, Continuous Renal Replacement Therapy; CKD, Chronic Kidney Disease; HR, Heart Rate; SBP, Systolic Blood Pressure; DBP, Diastolic Blood Pressure; MAP, Mean Arterial Pressure; CI, Cardiac Index; CPI, Cardiac Power Index; GEDI, Global End-Diastolic Volume Index; ELWI, Extravascular Lung Water Index; SVRI, Systemic Vascular Resistance Index; SVV, Stroke Volume Variation; PPV, Pulse Pressure Variation; PaCO₂, Partial Pressure of Arterial Carbon Dioxide; HCO₃⁻. Bicarbonate; Na, Sodium; HIRRT, Hemodynamic Instability Related to Renal Replacement Therapy. (a, Student’s t-test; b, Mann-Whitney U test; c, Fisher’s Exact test; d, Pearson Chi-square test)

## Discussion

In this prospective observational study, we comprehensively evaluated the incidence and associated factors of hemodynamic instability (HIRRT) in intensive care unit patients receiving continuous renal replacement therapy (CRRT). Our findings demonstrate that HIRRT is a significant cause of morbidity in this critical patient population and that hemodynamic parameters measured by transpulmonary thermodilution hold predictive value.

The HIRRT incidence of 55.5% identified in our study is notably higher than the 19–43% range reported in the literature [1, 10, 11]. This discrepancy may be primarily explained by the broader diagnostic criteria used in our study (a decrease in systolic blood pressure ≥ 20 mmHg or a decrease in mean arterial pressure ≥ 10 mmHg) and the more severe illness profile of our patient population [12]. Changes in vasopressor or inotropic requirements were not included in the diagnostic definition of HIRRT. Importantly, the absolute thresholds used to define HIRRT (≥ 20 mmHg systolic or ≥ 10 mmHg MAP decrease) may have different clinical implications in hypertensive versus normotensive patients; for example, a MAP decline from 90 to 80 mmHg may be physiologically well tolerated in some individuals. We acknowledge this as a limitation of using absolute cut-offs and note that future studies may benefit from incorporating relative changes or individualized thresholds based on premorbid hypertension status. The finding that 60% of HIRRT episodes occurred within the first hour of treatment highlights this period as a clinically critical phase, consistent with the study by Shawwa et al. [13], which reported a 65% incidence of hypotension within the first hour of CRRT.

An important finding of our study is that a CI < 2.61 L/min/m² appears to have predictive value for the development of HIRRT. This result supports the findings of Chazot et al. [14], who reported that a low CI is an independent risk factor for HIRRT. However, we found no significant association between preload parameters, such as the global end-diastolic volume index (GEDI), stroke volume variation (SVV), and pulse pressure variation (PPV), and HIRRT. This finding is partially inconsistent with the findings of Monnet et al. [3, 15] regarding the predictive value of preload dependence. This difference could be attributed to the heterogeneity of our patient population, variations in measurement protocols, or the fact that the vast majority of our patients (83.3%) were receiving vasopressor therapy, which may have affected the reliability of SVV and PPV. It should also be noted that PPV and SVV are not reliable predictors in patients with spontaneous breathing, which likely contributed to their lack of association with HIRRT in our cohort.

The finding that patients who developed HIRRT had higher baseline MAP and DAP may initially appear counterintuitive, but it is physiologically plausible. These patients also exhibited significantly higher baseline SVRI and lower CI, suggesting that the elevated MAP reflected compensatory vasoconstriction rather than hemodynamic robustness. Such a vasoconstrictive state may indicate limited vascular reserve, making patients more susceptible to the hemodynamic shifts triggered by CRRT. Moreover, because our definition of HIRRT relied on absolute thresholds for blood pressure decline, patients with higher starting MAP values may have been more likely to meet the criteria following modest relative decreases in pressure.

The depressive effects of uremic toxins on myocardial function have been demonstrated in experimental studies [16, 17]. Considering that 52.8% of our study population underwent CRRT due to uremic complications, it is plausible that a low CI may have contributed to the development of HIRRT in these patients. Furthermore, the significantly higher baseline SVRI in the group that developed HIRRT, and its lack of association with vasopressor doses (*p* = 0.135), suggests that uremia-induced dysregulation of vascular tone may also contribute to the pathogenesis. It is important to emphasize that while our data demonstrate associations between low CI, higher SVRI, and the presence of uremic indications, the proposed mechanisms involving myocardial depression and vascular tone dysregulation are speculative and based on prior literature rather than directly tested in this study.

Although SVRI showed a significant association with HIRRT in univariate analysis and is physiologically relevant, it did not remain an independent predictor in the multivariable model. Therefore, CI and MAP should be regarded as the primary independently predictive parameters in our cohort. The proposed cut-off values for CI and MAP should be considered preliminary and hypothesis-generating, given the wide confidence intervals and limited statistical power. Although these thresholds may help clinicians identify patients who require closer hemodynamic monitoring or more conservative ultrafiltration strategies, they are not ready for direct clinical implementation without validation in larger, multicenter cohorts.

Among the strengths of our study are its prospective design, standardized CRRT protocols, pre-treatment hemodynamic measurements, and comprehensive diagnostic criteria. However, factors such as its single-center nature, relatively small sample size, the lack of preload dependency tests, and the high mortality rate (75%) in the study population may limit the generalizability of the results.

This study has several limitations. First, it was conducted in a single center with a relatively small sample size, which may limit the generalizability of the findings. Second, the number of HIRRT events was limited (*n* = 20), which restricted the multivariable logistic regression model to only two predictors in order to reduce the risk of overfitting. Despite this restriction, the regression analysis should still be interpreted as exploratory, and the effect estimates may be unstable due to limited statistical power. Third, dynamic preload assessment tests (such as passive leg raising or fluid responsiveness measurements) were not performed prior to CRRT initiation, which prevented assessment of preload responsiveness and comparison of preload-dependent and preload-independent patients. Incorporating such standardized tests in future studies may help clarify the contribution of preload responsiveness to HIRRT development. In addition, dynamic changes in PiCCO-derived parameters (e.g., ΔCI or ΔSVRI during the first hour of CRRT) were not evaluated; incorporating time-series analyses in future research may provide further insights into the hemodynamic mechanisms underlying HIRRT. Finally, the exceptionally high in-hospital mortality rate (75%) indicates that the study population consisted of a highly critically ill and selected cohort, which may differ substantially from patient profiles in other ICUs. This high illness severity, combined with the single-center design, may limit external validity, as both the incidence of HIRRT and its associated risk factors could vary in less critically ill or more heterogeneous populations. Although no patients had known conditions that interfere with transpulmonary thermodilution, unrecognized or subclinical factors affecting measurement accuracy cannot be fully excluded.

In conclusion, our findings suggest that CI and SVRI may have clinical value in predicting the development of HIRRT during CRRT. Collectively, these results indicate that a reduced capacity to increase cardiac output in response to hemodynamic stress, together with alterations in vascular tone regulation, may play a central role in HIRRT development. Larger, multicenter prospective studies are needed to validate these findings and guide individualized treatment strategies. Additionally, further experimental studies investigating the effects of uremic toxins on the cardiovascular system may provide important insights into the mechanisms underlying HIRRT pathogenesis.

## Data Availability

The datasets used and/or analyzed during the current study are available from the corresponding author upon reasonable request.
